# The Relationship between Endothelial Dysfunction and Endothelial Cell Markers in Peripheral Arterial Disease

**DOI:** 10.1371/journal.pone.0166840

**Published:** 2016-11-18

**Authors:** Kimihiro Igari, Toshifumi Kudo, Takahiro Toyofuku, Yoshinori Inoue

**Affiliations:** Department of Surgery, Division of Vascular and Endovascular Surgery, Tokyo Medical and Dental University, Tokyo, Japan; Medical University Innsbruck, AUSTRIA

## Abstract

**Introduction:**

The endothelial function plays key roles in both promoting and protecting against atherosclerotic disease. Several methods, including peripheral arterial tonometry (PAT), have been reported to be useful for investigating endothelial dysfunction. Furthermore, the level of thrombomodulin (TM) is assumed to reflect the endothelial dysfunction. In the present study, we investigated endothelial dysfunction in patients with peripheral arterial disease (PAD) by measuring their TM levels, and evaluated the correlation between TM and various parameters.

**Materials and Methods:**

We measured the TM levels and performed PAT in 17 patients with PAD. We also recorded the patients’ demographic information, including comorbidities, and investigated their hemodynamic status by measuring the ankle brachial pressure index (ABI). The PAT results were used to calculate the reactive hyperemia index (RHI), which reflected the patients’ level of endothelial dysfunction.

**Results:**

The RHI and ABI values and the serum level of creatinine were found to be significantly correlated with the TM level (P = 0.040, 0.041 and 0.005, respectively). After setting an RHI value of 1.67 as the cut-off, the patients with RHI values of <1.67 were found to have significantly higher TM levels (median, 20.3 U/mL) than the patients with RHI values of ≥1.67 (median, 13.7 U/mL) (P = 0.023).

**Conclusions:**

The degree of endothelial dysfunction, as calculated by the TM level, might influence the prognostic value of the RHI, which is determined by PAT. The measurement of the TM level and PAT might both be useful methods of measuring endothelial dysfunction in patients with PAD.

## Introduction

The endothelium is a delicate monolayer of cells with whole blood vessels that plays an important role in the initiation and development of atherosclerotic diseases. Although the function of the endothelium is to protect against atherosclerotic disease, endothelial dysfunction can actually promote atherosclerotic diseases [1]. Furthermore, endothelial dysfunction plays key roles in vascular inflammation and thrombosis [2]. Thus, the measurement of the endothelial function has shown varying degrees of effectiveness in the stratification of atherosclerotic diseases [3].

Endothelial dysfunction has been established as an important early event in the pathogenesis of atherosclerosis, with changes in endothelial function occurring well before the onset of clinically apparent cardiovascular disease [4]. We can now evaluate the endothelial function by several non-invasive tests. Peripheral arterial tonometry (PAT) evaluates the digital pulse amplitude, which is a measurement of pulsatile volume changes [5]. Celermajer reported that PAT yields relevant predictive and prognostic data that reflect endothelial function [6]. PAT could therefore be used to assess changes in blood flow, which reflects the endothelial function—especially the microvascular endothelial function [7].

Furthermore, several plasma markers have shown a significant correlation with endothelial dysfunction [8, 9]. Thrombomodulin (TM), an endothelial membrane glycoprotein, plays a promising role in the protein C anticoagulant pathway. When TM, which is a thrombin receptor, is activated by thrombin, it facilitates protein C activation [10]. Some divided TM fragments exist in systemic circulation as soluble TM. Some studies have reported that the concentration of TM is associated with peripheral arterial disease (PAD) [11], and reflects the level of endothelial damage [12]. Sernau et al. concluded that TM is a marker of microvascular endothelial cell damage [13].

We conducted a pilot study to evaluate endothelial dysfunction in PAD patients by measuring their TM levels and performing PAT, and investigated the relationships between the TM level (as a measure of endothelial dysfunction) and various parameters.

## Material and Methods

### Patient selection

We recruited consecutive 17 patients with PAD who had undergone PAT and whose serum TM levels had been measured at an outpatient clinic at Tokyo Medical and Dental University Hospital between May 2014 and March 2015. PAD was diagnosed based on the presence of > 50% vessel stenosis due to lesions in the lower limbs. We mainly assessed the vessel stenosis by computed tomography angiography. In the cases with contraindication of using contrast media, such as chronic kidney disease and allergy, we evaluated the stenosis using magnetic resonance angiography and/or duplex ultrasound sonography. All of the patients provided their written informed consent, then we enrolled. This study was approved by the ethics committee of Tokyo Medical and Dental University (No. 701), and we reviewed the patients’ medical examinations and records. In the present study, we excluded patients who had recently undergone interventions for PAD or coronary artery disease (CAD) (< 6 months), and patients with a recent history (< 3 months) of myocardial infarction, unstable angina, decompensated heart failure, or cerebrovascular disease (CVD). We also excluded the patients with systemic inflammatory and malignant diseases. The patients’ medical records were reviewed as described below, and we investigated the patients’ demographics, medications and medical histories using a questionnaire. Hypertension was defined as a systolic blood pressure of > 130 mmHg, a diastolic blood pressure of > 80 mmHg or a history of treatment for hypertension. Dyslipidemia was defined as a serum low-density lipoprotein cholesterol level of > 140 mg/dL, a high-density lipoprotein cholesterol level of < 40 mg/dL, a triglyceride level of > 150 mg/dL or a history of treatment for dyslipidemia. CAD was defined as the presence of angina pectoris, myocardial infarction or both, on coronary angiography, or based on a history of the patient having undergone coronary artery revascularization. CVD was defined as a history of stroke, transient ischemic attack, carotid artery revascularization or cerebral hemorrhage. Diabetes mellitus was defined as having a fasting blood glucose level of > 126 mg/dL, a hemoglobin A1c concentration of > 6.5%, or the use of antidiabetic drugs. The severity of PAD was assessed by the measurement of the ankle brachial pressure index (ABI). The ABI was measured using a VasoGuard P84™ system (SciMed Ltd., Bristol, UK). We measured the systolic blood pressure on both sides of the brachial arteries in the upper limbs. In the lower extremities, both the posterior tibial arterial and dorsal pedis arterial pressures were measured if available. The ABI value was calculated based on the higher systolic blood pressure of the lower limbs divided by the higher brachial systolic blood pressure.

### Endothelial function test

To assess the endothelial function, pulse wave amplitude was assessed before and during reactive hyperemia, which is induced by occluding blood flow of brachial artery using inflatable cuff. The calculated index (reactive hyperemia index; RHI) between the flow in the upper arm with reactive hyperemia and the control upper arm represents a measure of the endothelial function [14]. We used the EndoPAT 2000 device (Itamar Medical Ltd., Caesarea, Israel), which is capable of determining the RHI, to measure the endothelial function. Patients refrained from smoking, drinking caffeine-containing beverages and eating food in the 12 hours before prior to PAT. The patients also stopped taking all drugs for 12 hours before the study. For PAT, the patients were placed in a supine position with a configured finger probe on the index finger of each hand, and a pressure cuff was placed on one upper arm, in a quiet room with a constant temperature of 20°C. The PAT signals from both fingers were continuously recorded during the 10-minute baseline period. The blood pressure cuff on the examined upper arm was then inflated to suprasystolic pressure for 5 minutes. The inflated cuff was then deflated, and the recording of the PAT signal continued for 10 minutes. Pressure changes reflecting the pulse amplitude both before the inflation of the cuff and after the deflation of the cuff, were transmitted to a computer, and the RHI was automatically calculated [15].

### Laboratory measurement

After the patients had fasted for at least 12 hours, non-traumatic venipuncture was performed in a superficial vein of upper limb to collect blood samples. Complete blood cell counts were measured and biochemistry tests were performed using standard laboratory methods. Several plasma aliquots were withdrawn (from the blood samples) after centrifugation in a floor-standing centrifuge for 20 minutes at 2,500 rpm. The samples were stored until use at -80°C. We also measured the serum levels of TM using a Thrombomodulin Enzyme Immunoassay kit (SRL, Inc., Tokyo, Japan).

### Statistical analysis

Categorical variables are expressed as frequencies and percentages, and continuous variables are expressed as median and interquartile range (IQR). The statistical significance was assessed using the Mann-Whitney *U* test for continuous variables. Correlations between the TM level and several parameters were assessed using Spearman’s correlation coefficient. P values of < 0.05 were considered to indicate statistical significance. The statistical analyses were performed using the Stat View software program (version 5, Abacus Concept Inc., Berkley, CA).

## Results

### Patient demographics

We evaluated 17 (14 males and 3 females) PAD patients in the present study. The median age was 71 years (IQR, 66–80), and the median body mass index was 22.0 kg/m^2^ (IQR, 20.8–22.8). The documented comorbidities included hypertension (82.4%), smoking history (70.6%), dyslipidemia (70.6%), DM (70.6%), CVD (29.4%) and CAD (17.6%). Ten patients (58.8%) were taking with Ca-blockers, and 3 patients (17.6%) were taking β-blockers. The laboratory test findings are shown in Table 1. With regard to the hemodynamic parameters, the median ABI level was 0.82 (IQR, 0.63–0.98), and the median RHI value (measured by PAT) was 1.46 (IQR, 1.36–1.68) (Table 1).

**Table 1:** Patients characteristics.

Variables	Subjects (n = 17)
Age (years), median, IQR*	71, 66–80
Gender (Male / Female)	14 / 3
Body mass index (kg/m^2^), median, IQR*	22.0, 20.8–22.8
Comorbidities	
Smoking history, number, percentage	12, 70.6%
Hypertension, number, percentage	14, 82.4%
Dyslipidemia, number, percentage	12, 70.6%
Coronary artery disease, number, percentage	3, 17.6%
Cerebrovascular disease, number, percentage	5, 29.4%
Diabetes mellitus, number, percentage	12, 70.6%
Medications	
Ca-blocker, number, percentage	10, 58.8%
β-blocker, number, percentage	3, 17.6%
Hemodynamical parameters	
Ankle brachial pressure index, median, IQR*	0.82, 0.63–0.98
Reactive hyperemia index, median, IQR*	1.46, 1.36–1.68
Laboratory findings	
White blood cell (/μl), median, IQR*	6400, 5700–7300
Hemoglobin (g/dl), median, IQR*	12.8, 12.2–14.7
Platelet (x10^4^/μl), median, IQR*	21.6, 18.9–25.2
Prothrombin time (%), median, IQR*	101.8, 91.7–107.3
Activated partial thromboplastin time (sec), median, IQR*	28.4, 27–30.1
Fibrinogen (mg/dl), median, IQR*	304, 269–317
D-Dimer (μg/ml), median, IQR*	1.06, 0.78–2.10
Creatinine (mg/dl), median, IQR*	0.92, 0.78–1.44
Total cholesterol (mg/dl), median, IQR*	193, 160–205
Triglycerides (mg/dl), median, IQR*	92, 71–205
High-density lipoprotein (mg/dl), median, IQR*	54, 48–67
Low-density lipoprotein (mg/dl), median, IQR*	116, 101–121
Hemoglobin A1c (%), median, IQR*	6.75, 6.45–7.05
C-reactive protein (mg/dl), median, IQR*	0.08, 0.05–0.17
Thrombomodulin (FU/ml)	18.5, 13.7–21.3

* *IQR*, interquartile range.

### Correlations between TM and other parameters

The correlations between TM and each of the parameters are shown in Table 2. None of the evaluated parameters, except the serum creatinine level and the RHI and ABI values, showed any significant correlation with the TM level. The TM level was significantly correlated with the serum creatinine level (ρ = 0.701, P = 0.005), and the RHI (ρ = -0.512, P = 0.040) and ABI (ρ = -0.510, P = 0.041) values. However, we evaluated the statistical significance by multiple testing with Bonferroni correction in which P values of < 0.0016 was considered to indicate statistical significance, then, serum creatinine, RHI and ABI did not show the statistical significant association with TM.

**Table 2:** The correlations between thrombomodulin and various parameters.

Variables	Correlation coefficient	P-value
Age	0.074	0.769
Gender	0.105	0.675
Body mass index	0.482	0.054
Comorbidities		
Smoking history	0.251	0.316
Hypertension	0.188	0.452
Dyslipidemia	0.398	0.144
Coronary artery disease	0.469	0.061
Cerebrovascular disease	0.230	0.358
Diabetes mellitus	0.240	0.337
Medications		
Ca-blocker	0.001	0.998
β-blocker	-0.093	0.710
Hemodynamical parameters		
Ankle brachial pressure index	-0.510	0.041
Reactive hyperemia index	-0.512	0.040
Laboratory findings		
White blood cell	0.213	0.395
Hemoglobin	-0.202	0.420
Platelet	-0.161	0.521
Prothrombin time	-0.425	0.090
Activated partial thromboplastin time	0.280	0.263
Fibrinogen	0.415	0.098
D-Dimer	-0.163	0.590
Creatinine	0.701	0.005
Total cholesterol	0.014	0.956
Triglycerides	-0.226	0.382
High-density lipoprotein	-0.124	0.642
Low-density lipoprotein	0.404	0.131
Hemoglobin A1c	-0.276	0.284
C-reactive protein	0.152	0.543

### The relationship between the TM level and the RHI and ABI values (S1 Table)

An RHI value of < 1.67 was defined as cut-off value of endothelial dysfunction [1]. We therefore divided the study population into two groups of RHI < 1.67 (n = 12) and RHI ≥ 1.67 (n = 5) according to their RHI values. The patients with RHI values of < 1.67 showed a significantly higher TM level (median, 20.3 U/mL) than patients with RHI values of ≥ 1.67 (median, 13.7 U/mL) (P = 0.023). Furthermore, we divided the patients who underwent a PAD test based on their ABI values of ≤ 0.90 (n = 11) and ABI > 0.90 (n = 6). The group patients with ABI values of > 0.90 showed a significantly lower TM level (median, 13.2 U/mL) than the patients with ABI values of ≤ 0.90 (median, 20.6 U/mL) (P = 0.004) (Table 3).

**Table 3:** Relationship between thrombomodulin and reactive hyperemia index, ankle brachial pressure index.

Variables (median, IQR*)	RHI^○^ < 1.67	RHI^○^ ≥ 1.67	P-value
Thrombomodulin	20.3 (18.1–27.4)	13.7 (12.6–14.3)	0.023
	ABI^△^ ≤ 0.90	ABI^△^ > 0.90	P-value
Thrombomodulin	20.6 (18.6–27.5)	13.2 (12.1–14.2)	0.004

* *IQR*, interquartile range

^○^ *RHI*, reactive hyperemia

^△^ *ABI*, ankle brachial pressure index.

## Discussion

TM is an endothelial membrane receptor for thrombin, which is an essential part of the protein C anticoagulant pathway. It has a large extracellular portion, in which most of its activity takes place [16]. Furthermore, some investigators have reported that TM may function as an antithrombotic and atheroprotective mediator [17]. Several factors have been reported to be correlated with the TM level, including inflammatory disease [18], systemic disease [19], and malignant disease [20]. Some types of atherosclerotic disease, including CAD [21], CVD [22] and aneurysmal disease [23], have been reported to be positively associated with TM. Furthermore, some studies have reported a positive association between TM and PAD [11, 24]. In line with the results of these previous studies, we showed a significant correlation between the TM level and the severity of PAD, as measured by ABI value; thus, the TM level might reflect the severity of PAD. We also found a significant correlation between the serum levels of TM and creatinine (ρ = 0.701, P = 0.005). Bao et al. [25] reported that serum TM level was significantly correlated with serum creatinine level (r = 0.778, P < 0.001), and TM was thought to play a key role in the development of chronic kidney disease.

We found a significant correlation between the TM level and the RHI value in the present study. The difference in blood flow, as measured by PAT, reflects microvascular dysfunction and might be useful for evaluating endothelial dysfunction at the microcirculation level [7] Several studies have reported a correlation between the TM level and PAD [24, 26]. Furthermore, the TM concentration could reflect the presence of microvascular complications and the severity of endothelial cell injury [27]. The present study found a significant correlation between the TM level and the RHI value (as measured by PAT), which have previously been reported as markers of microvascular endothelial dysfunction. To the best of our knowledge, this is the first study to report a positive association between the endothelial dysfunction, as measured by PAT and TM elevation.

The present study is associated with several limitations. First, the small sample size might have affected the statistical significance. We should therefore conduct a further study to confirm the significance of the correlations in a larger population. Second, our study included the PAD patients with relatively mild symptoms. The presence of PAD patients with critical limb ischemia or asymptomatic PAD patients in our population might have affected our results. Despite the limitations of the present study, we demonstrated significant correlations between the TM level (a measurement of endothelial dysfunction) and the RHI, as measured by PAT.

## Conclusions

We herein demonstrated that the TM level was significantly correlated with the RHI value, the ABI and the serum level of creatinine in PAD patients. Several factors might have affected our results. We should therefore perform a further study to confirm our findings. We used PAT as a novel method of evaluating the endothelial dysfunction of PAD patients. The results were confirmed based on the measurement of their TM levels, which showed the severity of endothelial dysfunction.

## Supporting Information

S1 Table Patients’ data of thrombomodulin and reactive hyperemia index, ankle brachial pressure index. (DOCX)
