# Supplementary material for: The Relationship between Endothelial Dysfunction and Endothelial Cell Markers in Peripheral Arterial Disease
Source: PLoS One. 2016 Nov 18;11(11):e0166840. doi: 10.1371/journal.pone.0166840 (PMC5115826; doi:10.1371/journal.pone.0166840)
Supplement: S1 Table — (DOCX) [file pone.0166840.s001.docx]

S1 Table. Patients’ data of thrombomodulin and reactive hyperemia index, ankle brachial pressure index.

| Patient No. | TM^□^ (FU/ml) | RHI^○^ | ABI^△^ |
| --- | --- | --- | --- |
| 1 | 27.2 | 1.28 | 0.84 |
| 2 | 20.6 | 1.23 | 0.79 |
| 3 | 27.8 | 1.36 | 0.60 |
| 4 | 12.6 | 1.46 | 0.79 |
| 5 | 11.4 | 1.68 | 0.97 |
| 6 | 18.3 | 1.93 | 0.98 |
| 7 | 11.9 | 1.37 | 0.98 |
| 8 | 16.7 | 1.55 | 0.58 |
| 9 | 21.3 | 1.61 | 0.90 |
| 10 | 28 | 0.96 | 0.63 |
| 11 | 19.9 | 1.43 | 0.59 |
| 12 | 13.7 | 1.72 | 0.98 |
| 13 | 18.5 | 1.13 | 0.54 |
| 14 | 18.7 | 1.36 | 0.82 |
| 15 | 12.6 | 1.73 | 1.02 |
| 16 | 14.3 | 1.75 | 1.09 |
| 17 | 33.1 | 1.53 | 0.78 |

^□^ *TM*, thrombomodulin; ^○^ *RHI*, reactive hyperemia; ^△^ *ABI*, ankle brachial pressure index.
